# Traitement des amyloses AL systémiques: à propos de 25 cas

**DOI:** 10.11604/pamj.2017.28.160.11885

**Published:** 2017-10-19

**Authors:** Hicham Eddou, Ali Zinebi, Hicham El Maaroufi, Mohammed Karim Moudden, Kamal Doghmi, Mohammed Mikdame, Mohammed El Baaj

**Affiliations:** 1 Service de Médecine Interne, Hôpital Militaire Moulay Ismail Méknes, Maroc; 2 Service d’Hématologie Clinique, Hôpital Militaire d’Instruction Mohammed V, Rabat, Maroc

**Keywords:** Amylose AL, Melphan-Dexamethasone, nouveau traitement, AL amyloidosis, melphalan-dexamethasone, new treatment

## Abstract

L'amylose AL systémique primitive est un désordre hématologique rare. La plupart des recommandations thérapeutiques sont basées sur des études de phase II ou des comparaisons rétrospectives et des séries de cas. Le but de cette étude était de décrire les cas d'amylose primitive AL et de faire une comparaison entre le protocole standard Melphlan-Dexamethasone et les nouveaux agents dans le traitement de première ligne de ces patients. Il s'agissait d'une étude rétrospective, descriptive et multicentrique, portant sur l'ensemble des cas d'amyloses AL colligées durant une période s'étalant de juillet 2009 à juin 2016 au sein de 2 centres hospitaliers militaires. Vingt cinq patients ont été colligés dans notre série (12 traités par le Melphalan-Dexamethasone et 13 par des protocoles contenant au moins du Bortézomib ou du Lénalidomide). Il n'y avait pas de différence significative entre les 2 groupes en termes de caractéristiques épidémiologiques, cliniques ou pronostiques. Après un suivi médian de 40 mois, la survie globale médiane était de 54 mois dans le groupe melphalan-Dexamethasone et de 60 mois dans le groupe nouvelles thérapeutiques (P = 0,98). Concernant la survie sans progression, elle était de 18 mois pour le groupe traitement standard contre 11 mois pour le 2^ème^ groupe (P = 0,08). Dans notre petite série nous n'avons pas trouvé une supériorité des nouvelles thérapeutiques par rapport au protocole classique. Ce résultat doit être confirmé par la réalisation d'une vraie étude prospective surtout en raison du coûtde ces nouvelles molécules qui ne sont pas toujours accessibles surtout dans les pays en voie de développement.

## Abstract (en)

Primarye systemic AL amyloidosis is a rare hematologic disorder. The majority of the therapeutic guidelines are based on phase II studies or on retrospective comparisons and case series. Our study aimed to describe all the cases of primary AL amyloidosis reported in 2 military hospitals and to make a comparison between standard melphalan-dexamethasone protocol and new agents in first-line treatment of patients with this disease. We conducted a retrospective, descriptive and multicentric study of all patients with AL amyloidosis whose data were collected during the period July 2009-June 2016. Twenty five patients were enrolled in the study (12 patients treated with melphalan-dexamethasone and 13 with bortezomib-based protocol or lenalidomide-based protocol). There was no significant difference in the epidemiological, clinical and prognostic features between the 2 groups. After a median follow up of 40 months, median overall survival was 54 months in the melphalan-dexamethasone-treated group and 60 months in the new therapies-treated group (P = 0.98). Progression-free survival was 18 months in the standard treatment group vs 11 months in the 2nd group (p = 0.08). In our small case series we haven’t found a superiority of the new therapies compared to the standard protocol. This result should be confirmed by a true prospective study, mainly because of the cost of these new molecules that are not always accessible, especially in developing countries.

## Introduction

L'amylose AL systémique primitive est un désordre hématologique en rapport avec une prolifération plasmocytaire (ou lymphoïde B) clonale produisant une chaine légère monoclonale d'immunoglobuline. Dans la plupart des cas, l'immunoglobuline monoclonale amyloïdogène peut être détectée dans le sérum et/ou les urines, soit par immunofixation, soit par le dosage des chaînes légères libres sériques. Ces protéines vont former des dépôts fibrillaires dans divers organes, ce qui conduit éventuellement à un dysfonctionnement organique et à la mort [1]. Le pronostic dépend du degré d'atteintes organiques et varie de quelques années à moins de 6 mois chez les patients atteints de cardiomyopathie grave [2]. L'objectif du traitement est la suppression du clone plasmocytaire anormal, ce qui conduit ensuite à une réduction des chaînes légères amyloïdogènes. La réponse hématologique se traduit généralement par une amélioration clinique des fonctions organiques et se combine avec un avantage de survie et une meilleure qualité de vie. Les régimes utilisés pour traiter les sujets atteints d'amylose systémique AL sont basés sur ceux utilisés dans le traitement du myélome multiple. L'amylose AL est une maladie rare et seules quelques études prospectives randomisées en phase III ont été menées. La plupart des recommandations sont basées sur des études de phase II ou des comparaisons rétrospectives et des séries de cas. Les choix thérapeutiques dépendent de l'âge du patient, de son état général et du type d'atteintes organiques [3]. Les stratégies thérapeutiques de première lignes pour la prise en charge des patients atteints d'amylose systémique AL comprennent l'intensification thérapeutique par Melphalan haute dose avec support de cellules souches hématopoïétiques autologues chez les patients éligibles pour un traitement intensif ou l'utilisation d'une chimiothérapie standard par le Melphan-Dexamethasone (MD) voire dans certains cas le recours aux nouvelles thérapies (inhibiteurs de protéasome, immunomodulateurs...) [4,5]. Le but de cette étude était de décrire les cas d'amylose primitive AL diagnostiqués dans 2 hôpitaux militaires et d'essayer de faire une comparaison entre le protocole standard MD et les nouveaux agents dans le traitement de première ligne de ces patients.

## Méthodes

### Patients

Cette étude multicentrique rétrospective type cas témoins a été mené au niveau de 2 centres hospitaliers et incluant les patients adultes (> 18 ans) porteur d'une amylose AL de novo avec une atteinte hématologique mesurable et une atteinte organique par dépôts amyloïdes. Le diagnostic d'amylose AL a fait appel à une biopsie tissulaire avec étude histologique montrant des dépôts amyloïde par coloration rouge du Congo, ainsi qu'une preuve de dyscrasie plasmocytaire (protéine monoclonale mesurable dans le sérum ou l'urine et/ou chaines légères sérique libre augmenté (> 100 mg/L) avec un rapport Kappa/Lambda anormal). Les critères d'atteinte organiques amyloïde ont fait appel aux recommandations du 10^ème^ symposium international sur les amyloses (Tableau 1) [6]. Ont été exclut de cette étude les patients présentant un myélome multiple symptomatique ou les cas d'amylose AL préalablement traités. Les patients ont été classés en 2groupes, selon le type du traitement reçu (MD Vs traitement par de nouveaux agents (contenant le bortézomib ou le lénalidomide)).

**Tableau 1:** Critères d’atteintes organiques dans l’amylose AL [6]

Organes	Critères d’atteinte
Cœur	Epaississement de la paroi cardiaque > 12 mm à l’échographie sans aucune autre cause **ou** NT pro BNP> 332 ng / l en l'absence d'insuffisance rénale
Rein	Protéinurie de 24 heures > 0,5 g/j, principalement l'albumine
Foie	Hépatomégalie > 15 cm en l'absence d’insuffisance cardiaque **ou** Phosphatase alcaline> 1 à 5 fois Limite supérieure de la normale
Gastro-intestinale	Vérification directe de la biopsie avec symptômes
Tissus mous	Macroglossie, Arthropathie, signes d’épaulette, Purpura périorbitaire Épaississement cutané confirmé par biopsie positive Myopathie par biopsie ou pseudo-hypertrophie Biopsie des ganglions lymphatiques positive Syndrome du canal carpien
Système nerveux	Atteinte périphérique clinique: neuropathie périphérique symétrique sensorimoteur des membres inférieurs. Atteinte dysautonomique: trouble de vidange gastrique, pseudo-obstruction, dysfonctionnement vésicale évocateur
Poumon	Signes respiratoires avec présence de dépôts amyloïdes sur biopsie Syndrome interstitiel à la radiographique pulmonaire

### Traitements

Les patients du groupe MD ont reçu des cours mensuels de melphalan oral (10 mg par mètre carré de surface corporelle aux jours 1 à 4) plus de la dexaméthasone par voie orale (40 mg par jour aux jours 1 à 4) pendant 6 à 12 mois en fonction de la réponse hématologique, en général 3 mois après la réponse complète ou l'obtention d'un plateau. Une Prophylaxie par inhibiteurs de pompe à proton et triméthoprime-sulfaméthoxazole (Un comprimé trois fois par semaine) a été administrée dans la majorité des cas. Pour le 2^ème^ groupe 4 protocoles ont été utilisé contenant au moins le lénalidomide ou le bortézomib à savoir: Velcade- Dexaméthasone (VD), Velcade-Melphalan- Dexaméthasone (VMD), Velcade-Cyclophosphamide-Dexaméthasone (CyBorD) et le Revlimide- Dexaméthasone (Rd). Concernant le protocole VD, le bortézomib a été administré par voie intraveineuse à la dose standard de 1,3 mg/m^2^ aux jours 1, 4, 8 et 11 plus la dexaméthasone par voie orale (40 mg par jour aux jours 1 à 4 et 9 à 12) avec des cycles tous les 21 jours. Pour le protocole VMD, le bortézomib a été administé à la même dose mais d'une manière hebdomadaire, alors que le Melphalan et la Dexamethasone suit le protocole MD classique. Les patients sous protocole CyBorD ont reçu le bortézomib et la dexaméthasone suivant le protocole VD associé au cyclophosphamide à la dose de 300 mg/m^2^ aux jours 1, 8, 15, et 22. Enfin pour le protocole Rd, le Lénalidomide a été administré à la dose de 25mg/j du 1^er^ au 21^ème^ jour et la Dexaméthasone à la dose de 40 mg/j (aux jours 1 à 4, 9 à 12 et 17 à 20) avec des cycles tous les 28 jours.

### Evaluations

L'examen physique, les paramètres de laboratoire (hémogramme, bilan hépatique, calcémie, créatinémie, protéinurie, électrophorèse des protéines sériques ou dosage des chaines légères libres sériques) ont été obtenus mensuellement. L'indice de performance (Performence status) a été évalué comme décrit par les critères de l'ECOG (eastern cooperative oncology group). L'évaluation pronostique des patients atteints d'amylose AL a fait appel au score pronostique de la Mayo clinique basé sur la NTproBNP et la troponine [7]. La toxicité thérapeutique a été enregistrée selon les critères du National Cancer Instiute (Cancer Therapy Evaluation Program, Common Terminology Criteria for Adverse Events (Version 3.0)).

La réponse au traitement (hématologique et organique) a été évalué selon les recommandations du consensus internationales (Tableau 2) [6,8,9]. Les réponses hématologiques et cliniques ont été évaluées chez les patients qui ont reçu au moins trois cures du traitement. Une réponse clinique a été définie comme l'amélioration d'une atteinte organique initiale rattachée à l'amylose. Une réponse hématologique complète a été définie comme la disparition complète de l'immunoglobuline monoclonale ou de la chaîne légère dans un échantillon de sérum ou d'urine; Une réponse hématologique partielle a été définie comme une réduction de plus de 50% de ces protéines.

**Tableau 2:** Critères de réponse et de progression des hématologique et organique dans l’amylose AL [6,8, 9]

	**Réponse**	**progression**
Hématologique	**Réponse complète (CR)** Absence de composant monoclonal sérique et urinaire avec rapport kappa/lambda normal. **Très bonne réponse partielle (VGPR)** Différence de concentration des chaines légères libres (dFLC) < 40mg/L. **Réponse partielle (PR)** Diminution de la dFLC≥50 %. **Absence de réponse** Toute autre situation.	**Depuis une CR** Tout composant monoclonal détectable ou rapport kappa/lambda anormal avec doublement de la chaine légère incriminée. **Depuis une PR** Augmentation de 50% de la protéine monoclonale sérique (au moins 5g/l) Augmentation de 50% de la protéine monoclonale urinaire (au moins 200mg/24h avec un pic visible) Augmentation de 50% des chaines légères libres (au-delà de 100mg/l)
Cœur	Diminution de 2mm de l’épaisseur du septum inter ventriculaire Augmentation de 20% de la fraction d’éjection ventriculaire Amélioration de 2 points de la classification de la NYHA sans augmentation des diurétiques ni augmentation de l’épaisseur du septum inter ventriculaire Réduction (≥ 30% ou ≥ 300ng/l) du NT-pro BNP chez les patients ayant une clairance de la créatinine ≥ 45ml/min/1.73m^2^	Augmentation de 2mm du septum inter ventriculaire Augmentation d’1 point de la classification NYHA associé à une diminution de la fraction d’éjection ≥ 10%
Rein	Diminution de 50% (au moins 0,5g/24h) de la protéinurie des 24h sans diminution de plus de 25% de la clairance de la créatinine ni augmentation de plus de 25% de la créatinine sérique	Augmentation de 50% de la protéinurie des 24h (doit être > à 1g/24h) Aggravation de 25% de la clairance de la créatinine ou de la créatinine sérique
Foie	Une diminution ≥ 50% des taux de phosphatase alcaline et / ou Diminution ≥ 2 cm de la taille du foie (évaluée par imagerie)	augmentation ≥ 50% des taux de phosphatase alcaline de la plus basse valeur enregistrée
Système nerveux périphérique	Amélioration de la vitesse de conduction nerveuse à l'électromyogramme	Neuropathie progressive à l’électromyogramme

### Méthodes

Il s'agissait d'une étude rétrospective, descriptive et multicentrique, portant sur l'ensemble des amyloses AL colligées durant période s'étalant de juillet 2009 à juin 2016 au sein de centres hospitaliers: Service de médecine interne de l'hôpital Moulay Ismaïl à Meknès, Maroc; Service d'Hématologie Clinique, Hôpital Militaire d'instruction Mohammed V Rabat Maroc.

### Analyses statistiques

Les données quantitatives ont été présentées en médiane et les données qualitatives sont rapportées en pourcentage. Les courbes de survie ont été déterminées avec la méthode de Kaplan Meier et le test du log-rank était utilisé pour comparer les variables. L'analyse statistique a été réalisée avec le logiciel SPSS version 20.0 au niveau du laboratoire de biostatistique de la faculté de médecine de Fès (Maroc).

## Résultats

### Caractéristiques généraux (Tableau 3)

Entre Juillet 2009 et le Juin 2016, 25 patients présentant une amylose AL primitive de novo ont été admis au niveau des 2 centres. Le Tableau 3 résume les principales caractéristiques de la population des deux groupes. Le type d'amylose AL a été déterminé par analyse immunohistochimique pour l'ensemble des cas. Tous les patients présentaient une protéine monoclonale au niveau du sang ou des urines. Les caractéristiques de la maladie amyloïde étaient bien équilibrées entre les deux groupes de patients. Au total, L'âge médian au diagnostic était de 64,6 ans avec un sex ration hommes pour femmes de 1,5. L'évaluation de l'état général selon l'ECOG notait que 52 % des patients avaient un score de 2 et 3 (50% pour le 1^er^ groupe et 53% pour le second). Concernant les atteintes organiques, 13 patients avaient une atteinte cardiaque (8 pour le 1^er^ groupe et 5 pour le second), 16 patients avaient une atteinte rénale (7 pour le 1^er^ groupe et 9 pour le second), 9 patients avaient une atteinte digestive (4 pour le 1^er^ groupe et 5 pour le second) et 6 patients avaient une atteinte hépatique (5 pour le 1^er^ groupe et 1 pour le second). 44 % des patients avaient une atteinte de plus de 2 organes (50% pour le 1^er^ groupe et 39% pour le second). En utilisant la classification de la Mayo Clinic, treize de nos patients entraient en stade III (67% pour le 1^er^ groupe contre seulement 38% pour le second).

**Tableau 3:** Les principales caractéristiques des 25 patients de notre série

Variable	Total	Groupe traitement classique	Groupe nouveau traitement	Signification p
**Age** (moyenne)	64,6 +/- 12,1	65,58 +/- 10,8	63,7 +/- 13,6	0,70
**délai diagnostic** (mois)				0,67
Médiane	5,88 +/- 3,33	5,58 +/- 3,2	6,15 +/- 3,55	
**Sexe**				
Homme	15	7	8	
Femme	10	5	5	
Sex ration (H/F)	1,5	1,4	1,6	0,8
**Indice de Performance (n)**				0,76
0	2	0	2	
1	9	6	3	
2	9	4	5	
3	4	2	2	
**Score pronostic**				0,56
I	12	4	8	
II	0	0	0	
III	13	8	5	
**Nombre d’organe atteint**				0,64
1	9	3	6	
2	5	3	2	
3	7	3	4	
4	4	3	1	
**Les organes atteints :**				
Cœur	13	8	5	
Rein	16	7	9	
Digestive	9	4	5	
Foie	6	5	1	
Articulaire	3	1	2	
Système nerveux	2	1	1	
**Données biologiques :**				
BNP>120 pg/ml	12	7	5	
Beta 2 microglobulin >3.5 mg/l	9	3	6	
Créatinine >2 mg/dl 3	5	2	3	
Albumine >3.5 g/dl	11	5	6	

### Réponse au traitement (Tableau 4)

Le taux de réponse global était de 56% (14 de 25 patients), huit patients atteignant une RC ou une TBRP hématologique (32%) et six autres patients atteignant une RP (24%). Comme le montre le Tableau 4. Il n'y avait pas de différence significative entre les 2 groupes (58% de réponse pour le groupe MeL-Dex et 54% pour les patients recevant les nouveaux traitements). Il est à noter qu'un seul patient a connu une progression de la maladie. Concernant la réponse clinique, Une amélioration de la fonction organique a été observée chez 10 (40%) patients dont 4 dans le groupe du traitement standard et 6 pour le groupe des nouvelles thérapeutiques.

**Tableau 4:** Réponse au traitement (hématologique et clinique)

Variable	Total	Groupe traitement classique	Groupe nouveau traitement
**Réponse hématologique**			
Rémission complète + Très bonne réponse partielle	8 (32%)	4 (33%)	4 (31%)
Réponse partielle	6 (24%)	3 (25%)	3 (23%)
Pas de réponse	6 (24%)	2 (17%)	4 (31%)
Progression	5 (20%)	3 (25%)	2 (15%)
**Réponse clinique**	10 40%)	4 (33%)	6 (46%)

### La survie (Figure 1, Figure 2)

Le suivi médian des patients était de 42 mois. La survie médiane globale pour l'ensemble de la série était de 55 mois sans différence entre les 2 groupe (54 mois pour le groupe Mel-Dex et 60 pour le groupe nouveau traitement) (P = 0,96). Concernant la survie sans progression, elle était de 12 mois avec un avantage pour le groupe traitement standard (18 mois) par rapport au nouveau traitement (11 mois) mais qui reste non significative (P = 0,08). Parmi les 14 décès enregistré dans notre série, un seul était lié à une toxicité directe du traitement (choc septique chez un patient traité par Mel-Dex). Pour les autres, le décès étaient secondaire à la progression de la maladie (essentiellement par décompensation cardiaque).

**Figure 1:**
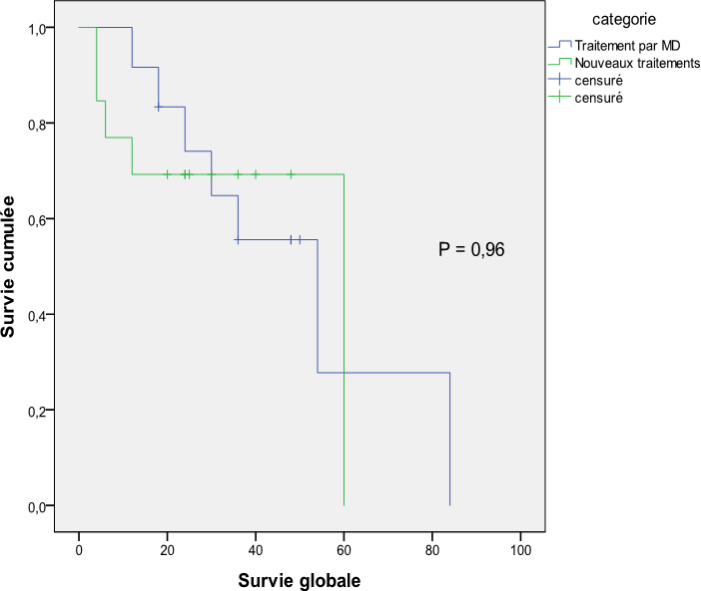
Survie globale en fonction du type de traitement

**Figure 2:**
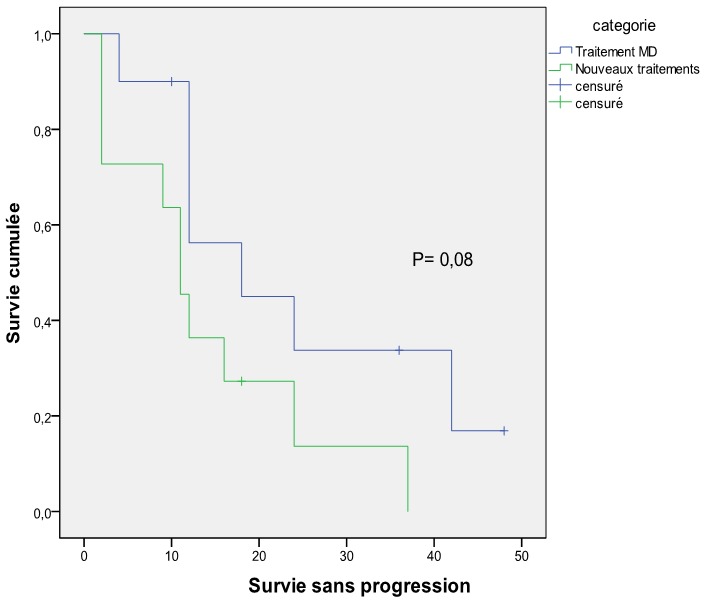
Survie sans progression en fonction du type de traitement

## Discussion

Cette étude rétrospective menée au niveau de 2 hôpitaux militaires d'une série de 25 patients présentant une amylose AL systémique avait pour but principale de comparer l'apport des nouvelles thérapies dans cette pathologie par rapport au traitement historique qui est le MD. L'amylose AL est une pathologie rare avec une incidence annuelle aux alentours de 500 cas en France. Elle touche un peu plus les hommes que les femmes avec un âge moyen au diagnostic de 65 ans [10]. L'amylose systémique est liée à la présence de chaînes légères toxiques qui forment des dépôts fibrillaires extracellulaires provoquant un dysfonctionnement progressif des organes vitaux et éventuellement la mort, principalement de causes cardiaques. Le diagnostic fait appel à la coloration au rouge du Congo d'un échantillon de biopsie tissulaire, qui présente un dichroïsme pathognomonique vert pomme sous lumière polarisée [11]. Les organes les plus fréquemment touchés par l'amylose AL au diagnostic sont le cœur (50%), les reins (50%), le foie et le tractus gastro-intestinal (25%) et les nerfs périphériques (20%) [6]. Une atteinte organique multiple est courante au moment du diagnostic comme c'était le cas dans notre série ou presque la moitié des cas avaient une atteinte d'au moins trois organes. Une évaluation approfondie doit être effectuée, comprenant une histoire détaillée, un examen physique complet, des tests de laboratoire et des études d'imagerie cardiovasculaire. Le score pronostic cardiaque (se basant sur Le NT-proBNP et la troponine) reste le plus utilisé en pratique courante et dans le cadre d'essai clinique. Les survies médianes pour les stades I, II et III sont de 26,4; 10,5 et 3,5 mois respectivement [12]. L'augmentation de ces bio-marqueurs indique souvent la progression de la maladie, avec 2 exceptions possibles à savoir l'insuffisance rénale et le traitement par immunomodulateurs (Notamment le lenalidomide) qui peuvent entraîner des niveaux élevés aussi [13].

Bien que l'amylose AL demeure une maladie souvent incurable, beaucoup de progrès ont été réalisés au cours de ces dernières années aidé par l'élaboration et la validation des critères d'atteintes organiques, de stadification pronostic et de réponses aux traitements (hématologique et clinique). Dans l'amylose AL, les études prospectives randomisées de phase III sont rarement menées, et la plupart des recommandations reposent sur des études de phase II ou des comparaisons rétrospectives et des séries de cas. La chimiothérapie est basée sur des schémas utilisés pour le traitement du myélome, avec des adaptations en termes de dose et de calendrier et qui ont pour but de stopper la production des chaines légères libres pathologiques qui se traduit par une amélioration significative de la survie pour la plupart des patients [14]. Le traitement à base d'alkylant, que sa soit à dose standard ou élevée (dans le cadre d'une intensification thérapeutique) a été le pilier de la thérapie pendant de longues décennies. Le schéma MD classique est associée, comme c'est le cas de notre série, à des résultats favorables tant sur la réponse hématologique et clinique (67% et 48% respectivement) que sur la survie (4 ans en moyen) [15]. Mais les meilleures résultats était retrouvé essentiellement chez les patients avec une maladie à risque faible ou intermédiaire; Cependant, chez les patients à haut risque, les résultats du traitement par MD étaient médiocres [16, 17]. Et bien qu'il n'y ait pas eu de comparaison directe dans les essais cliniques, des protocoles à bases de Bortézomib (notamment le CyBorD) ont rapidement remplacé le MD en tant que traitement standard chez les patients non éligibles à une intensification thérapeutique et présentant une atteinte cardiaque au stade III [18, 19]. Cependant dans notre série où les formes avancées (stade III et nombre d'organes atteint) étaient plus fréquentes dans le groupe MD que le groupes nouvelles thérapies on ne trouvait pas de différences significative entre les 2 groupes tant en matière de réponse, toxicité ou survie.

## Conclusion

Le traitement des amyloses AL systémiques constitue un chalenge aussi bien pour les praticiens que pour les patients. Plusieurs nouvelles molécules prennent la place du traitement classique MD essentiellement dans les stades avancées sans qu'il y ait une étude prospective de phase III les comparants. Dans notre petite série nous n'avons pas trouvé une supériorité de ces nouvelles thérapeutiques pouvant explique leur utilisation. Mais la confirmation de ce résultat nécessite la réalisation d'une vraie étude prospective surtout en raison du coût de ces molécules qui ne sont pas toujours accessibles surtout dans les pays en voie de développement.

### Etat des connaissances actuelle sur le sujet

- L'amylose AL est une hémopathie maligne de pronostic réservé;
- Le traitement fait appel à une chimiothérapie classique type Melphalan-Dexamethasone ou des thérapies ciblées pour les formes de bon pronostic alors que pour les formes avancées seules ces dernieres sont recommandés.

### Contribution de notre étude à la connaissance

- L'analyse des résultats de notre série ne trouve pas de différence significative entre les 2 schémas thérapeutiques même dans les formes de mauvais pronostic.

## Conflits d’intérêts

Les auteurs ne déclarent aucun conflit d'intérêts.
